# High-Throughput Imaging of CRISPR- and Recombinant Adeno-Associated Virus–Induced DNA Damage Response in Human Hematopoietic Stem and Progenitor Cells

**DOI:** 10.1089/crispr.2021.0128

**Published:** 2022-02-22

**Authors:** Daniel Allen, Lucien E. Weiss, Alon Saguy, Michael Rosenberg, Ortal Iancu, Omri Matalon, Ciaran Lee, Katia Beider, Arnon Nagler, Yoav Shechtman, Ayal Hendel

**Affiliations:** ^1^ Institute of Nanotechnology and Advanced Materials, The Mina and Everard Goodman Faculty of Life Sciences, Bar-Ilan University, Ramat-Gan, Israel; ^2^ Department of Biomedical Engineering, Technion, Haifa, Israel; ^3^ Department of Engineering Physics, Polytechnique Montreal, Canada; ^4^ Arazi School of Computer Science, Interdisciplinary Center, Herzliya, Israel; ^5^ APC Microbiome Ireland, University College Cork, Cork, Ireland; ^6^ Division of Hematology and Bone Marrow Transplantation, Chaim Sheba Medical Center, Tel-Hashomer, Ramat Gan, Israel; ^7^ Sackler Faculty of Medicine, Tel Aviv University, Tel Aviv, Israel.

## Abstract

CRISPR-Cas technology has revolutionized gene editing, but concerns remain due to its propensity for off-target interactions. This, combined with genotoxicity related to both CRISPR-Cas9-induced double-strand breaks and transgene delivery, poses a significant liability for clinical genome-editing applications. Current best practice is to optimize genome-editing parameters in preclinical studies. However, quantitative tools that measure off-target interactions and genotoxicity are costly and time-consuming, limiting the practicality of screening large numbers of potential genome-editing reagents and conditions. Here, we show that flow-based imaging facilitates DNA damage characterization of hundreds of human hematopoietic stem and progenitor cells per minute after treatment with CRISPR-Cas9 and recombinant adeno-associated virus serotype 6. With our web-based platform that leverages deep learning for image analysis, we find that greater DNA damage response is observed for guide RNAs with higher genome-editing activity, differentiating even single on-target guide RNAs with different levels of off-target interactions. This work simplifies the characterization and screening process of genome-editing parameters toward enabling safer and more effective gene-therapy applications.

## Introduction

Many sequence-recognition systems have been utilized for gene editing.^1–5^ However, the easily programmable nature of the CRISPR-Cas system makes it particularly advantageous. The successful application of CRISPR-Cas complexes, the most popular of which is Cas9, to gene editing in human cells has highlighted the system's promise as a therapeutic tool in the context of genetic disorders.^6–8^

The Cas9 nuclease induces a double-strand break (DSB) at a DNA target site with a sequence complementary to that of a programmed guide RNA (gRNA). Repair proteins detect the break sites and activate the DNA damage response (DDR) pathway, triggering the recruitment of other repair machinery to the break site via the phosphorylation of H2A histone family member X (H2AX) to γH2AX. The signal is then subsequently amplified over megabasepairs of nearby chromatin.^9,10^ This forms a multi-unit chromatin structure that clusters around a single DSB site facilitating its repair.^11^ γH2AX immunolabeling has been shown to be a robust marker for visualizing DDR and genotoxicity. Repair-center clusters manifest as foci under the microscope,^12,13^ facilitating quantification of DNA damage caused by gamma irradiation, zinc-finger nucleases (ZFNs),^14,15^ and extremely promiscuous CRISPR gRNAs.^16^

In its native context, CRISPR-Cas functions as a defense mechanism to cleave foreign DNA in bacteria and archaea. There, some flexibility to recognize sequence variants is advantageous.^17,18^ In the context of gene therapies, however, the result of nonspecific DNA damage can be catastrophic.^19^ If left unrepaired, even a small number of DSBs are sufficient to cause cell-cycle arrest and genotoxic downstream effects such as breaks in mitosis, aneuploid progeny, and cell death.^20^

CRISPR-induced DSBs can be repaired by either non-homologous end joining (NHEJ) or homology-directed repair (HDR). NHEJ can result in insertions or deletions (indels), causing frameshift mutations that can be used to knock out target genes, while HDR can be used to correct target genes via introduction of an exogenous donor DNA template. Accumulation of off-target mutations, such as insertions, deletions, and chromosomal aberrations, can lead to apoptosis, cell senescence, and the onset of devastating diseases such as cancer. While significant progress has been made to minimize CRISPR-Cas-induced unintended damages,^21–25^ this system is not yet an error-free tool.

Apart from sequence homology, which can be easily computed using *in silico* tools, the propensitiy for off-target activity is difficult to predict due to the strong influence of biochemical factors in the cellular environment, thus necessitating experimental measurement assays. Existing experimental methods for detecting off-target activity include *in vivo*/cell-based workflows such as genome-wide, unbiased identification of DSBs enabled by sequencing (GUIDE-seq)^26^ and discovery of *in situ* Cas off-targets and verification by sequencing (DISCOVER-seq),^27^ as well as a suite of *in vitro*/cell-free methods such as circularization for high-throughput analysis of nuclease genome-wide effects by sequencing (CHANGE-seq),^28^ and selective enrichment and identification of adapter-tagged DNA ends by sequencing (SITE-seq).^29^

We recently demonstrated a combined pipline using GUIDE-seq and RNase H2-dependent multiplex assay amplification (rhAmpSeq)^30,31^ to quantify off-target activity with next-generation sequencing (NGS). While the off-target information provided by sequencing is important, this represents an “end-product” of CRISPR-Cas activity and does not provide detailed genotoxicity charecterization based on DDR induction. Characterizing the DDR dynamics can therefore fill an important knowledge gap for minimizing the genotoxic effects induced by numerous genome-editing reagents such as engineered nucleases or donor DNA vectors.

Other than CRISPR-induced DDR, an additional aspect of genome editing that can lead to genotoxicity is viral-based donor-DNA delivery. One of the most promising methods used in HDR-related genome editing employs recombinant adeno-associated virus serotype 6 (rAAV6) for donor DNA delivery, which has been shown to trigger a DDR that is measurable through γH2AX staining.^32,33^ The AAV-induced DDR triggers p53 activation, inhibits human hematopoietic stem and progenitor cell (HSPC) proliferation, and promotes cell death and apoptosis via immune detection.^34^ Therefore, there is a need to develop a mechanism-independent, inexpensive, and rapid tool to optimize protocols by minimizing genome-editing-related genotoxicity through assessment of the DDR.

In this study, we employ imaging flow cytometry (IFC) to evaluate and characterize the extent of the DDR following CRISPR-Cas9 and rAAV6 treatment on the single-cell level. IFC combines the subcellular spatial information obtained by microscopy^35^ and the high throughput of flow cytometry. This facilitates the collection of large data sets containing thousands of individual cell images to characterize the DDR.^36,37^

Our method, available in a web-based platform, uses deep learning and localization microscopy to quantify fluorescently labeled γH2AX puncta in images from which we assess the amount of DNA damage in a particular treatment. We show that (1) this method is applicable to gRNAs with multiple on-target sites; (2) our approach is sufficiently sensitive to detect differences between multiple gRNAs with a single on-target site; and (3) rAAV6 transduction conditions that achieve therapeutically relevant levels of HDR can be optimized to minimize DNA damage. This pipeline represents a strategy to expand the range of tested conditions significantly. In concert with off-target NGS analysis, this approach will facilitate comprehensive optimization of genome-editing parameters for gene-therapy application.

## Methods

### Cells and cell culture conditions

Cord blood (CB)-derived CD34^+^ HSPCs were obtained from the Sheba Medical Center CB bank under Institutional Review Board–approved protocols to obtain CB units for research purposes. CD34^+^ HSPCs were isolated using magnetic beads (Miltenyi) following the conventional method. CD34^+^ HSPCs were cultured at 37°C, 5% CO_2_, and 5% O_2_ and in SFEM II supplemented with 100 ng/mL Flt3-Ligand, 100 ng/mL thrombopoietin (TPO), 100 ng/mL stem-cell factor (SCF), 0.035 mg/μL UM171, 0.75 mg/μL SR1 (Stemcell Technologies), 20 IU/mL penicillin, and 20 mg/mL streptomycin (Biological Industries).

HEK293 cells with stable expression stable expression of Cas9 (HEK293-Cas9) were provided by Integrated DNA Technologies (IDT) and were cultured in Dulbecco's modified Eagle's medium supplemented with 10% fetal bovine serum, 0.1 mM NEAA, 6 mM l-glutamine, 1 mM sodium pyruvate, and 1% penicillin/streptomycin (Biological Industries). Additionally, 500 μg/mL G418 (Gibco Invitrogen) was added for neomycin-resistant cell selection. Cells were incubated at 37°C and 5% CO_2_ and were allowed to grow to 70–80% cell confluency.

### CRISPR-Cas9 preparation and nucleofection

CRISPR-Cas9 preparation and nucleofection was conducted in accordance with Shapiro *et al*.^38^ A modified synthetic single guide RNA (sgRNA; Alt-R^®^ CRISPR-Cas9 sgRNA; IDT) was precomplexed with Cas9 protein (Alt-R^®^ S.p. Cas9 Nuclease V3; IDT) at a molar ratio of 1:2.5 (Cas9 to gRNA) for 20 min at 25°C to form the ribonucleoprotein (RNP) complex (see Table 1 for gRNA sequences). CD34^+^ HSPCs were reconstituted in P3 primary cell electroporation solution according to the manufacturer's instructions (Lonza) and mixed with RNP complexes at a final concentration of 4 μM. The supplemented cell solution (final cell concentration of 2 × 10^6^ cells/mL) was transferred into the Lonza 4D-Nucleofector and electroporated using the DZ-100 program.

**Table 1.** gRNA Sequences and the Number of On-target Sites

Name of gRNA	Sequence (5′→3′)	Number of on-target sites
gRNA_9(i)_	TCTGAAAGTGCTGGGATCAC	9
gRNA_9(ii)_	GCATGTGCCACCACCATGCC	9
gRNA_9(iii)_	CATCTGTAATCTCAGCAATT	9
gRNA_9(iv)_	ATCTGTAATCTCAGCAATTT	9
gRNA_105_	TTGCCCAAGCTAGAGTGCAA	105
gRNA_1,055_	CTCAGGTGATCCACCCTCCT	1,055
gRNA_22,606_	CAGGAGAATCGCTTGAACCT	22,606
*RAG1*	AACTGAGTCCCAAGGTGGGT	1
*RAG2*	TGAGAAGCCTGGCTGAATTA	1
*VEGFA*	GGGTGGGGGGAGTTTGCTCC	1

For the GUIDE-seq experiments, HEK293-Cas9 cells were electroporated with two-part gRNA system comprised of CRISPR RNA (Alt-R^®^ CRISPR-Cas9 crRNA XT; IDT) annealed to a transactivating CRISPR RNA molecule (Alt-R^®^ CRISPR-Cas9 tracrRNA; IDT).

### rAAV6-based DNA donor design and vector production

All homology-based rAAV6 vector plasmids were designed and cloned into pAAV-MCS plasmid (Agilent Technologies, Santa Clara, CA) containing AAV2-specific inverted terminal repeats. The donor DNA was designed with 400 bp left and right arms of homology flanking the gRNA cut site. The donor DNA included a tNGFR reporter gene under the control of a PGK promotor and *RAG2* cDNA for gene correction. The pDGM6 plasmid, containing the AAV6 cap genes, AAV2 rep genes, and adenovirus helper genes, was received from David Russell (University of Washington). The final rAAV6 vector was produced by Vigene Biosciences in large-scale rAAV6 packaging (Vigene Biosciences).

### Genome targeting and quantification

CD34^+^ HSPCs were electroporated with the *RAG2* sgRNA/Cas9 RNP system and were seeded at a density of 0.33 × 10^6^ cells/mL. In HDR experiments, the cells were immediately transduced with the rAAV6 donor at multiplicities of infection (MOIs) of 200, 2,000, and 20,000 viral genomes (VG)/cell within 5 min of electroporation. Cells were cultured at 37°C, 5% CO_2_, and 5% O_2_ in SFEM II supplemented with 100 ng/mL Flt3-Ligand, 100 ng/mL TPO, 100 ng/mL SCF, 20 IU/mL penicillin, 20 mg/mL streptomycin, 0.035 mg/μL UM171, and 0.75 mg/μL SR1 (Stemcell Technologies) for the necessary time period after which they were taken for analysis in the Amnis^®^ ImageStream^®^ XMk II (Amnis) or by standard flow cytometry performed in the BD LSRFortessa™ (BD Biosciences) or the Accuri C6 flow cytometer (BD Biosciences).

### Immunostaining

After CRISPR-Cas9 edited cells were incubated in culture medium at 37°C with 5% CO_2_ and 5% O_2_, cells were centrifuged at 400 *g* for 7 min, washed with phosphate-buffered saline (Biological Industries), and centrifuged again. Supernatant was then removed, and the cell pellet was suspended in 1 mL freshly prepared 2% paraformaldehyde (PFA) from Pierce™ 16% formaldehyde (w/v) methanol-free ampules (Thermo Fisher Scientific). Fixation in PFA was performed for 10 min at room temperature. Cells were then centrifuged at 400 *g* for 7 min. The supernatant was removed and stored in 70% ethanol at −20°C overnight.

Cells were then centrifuged at 400 *g* for 7 min and rehydrated and permeabilized in 0.1% Triton X-100 (Sigma–Aldrich) and 3% bovine serum albumin (MP Bio) for 30 min at room temperature. Cells were stained at a dilution of 1:1,000 with AF488 MS anti-γH2AX (PS139; clone: N1-431; BD Pharmingen) and incubated at room temperature for 1 h. Cells were washed and centrifuged at 400 *g* for 7 min. Volume was reduced to ∼70 μL and stained with DAPI where noted (10 μg/mL) (Sigma–Aldrich). Cells were read in the Amnis^®^ ImageStream^®^ XMk II (Amnis). Standard flow cytometry analysis was performed in the BD LSRFortessa™ (BD Biosciences) and the Accuri C6 flow cytometer (BD Biosciences).

### IFC

Samples were vortexed and loaded into the Imagestream. Laser intensity for the 488 nm was set to 150 mW, and the 405 nm was set to 10 mW in situations where DAPI staining was used. Cells were imaged at 60 × magnification. Typical runtime was several minutes per sample and was limited by cell density.

To remove images that contained multiple cells or cell debris or which were out of focus, only cells that met the following four criteria were included in the analysis: Area_M06 > 175; MeanPixel_M06_Ch06>-20; GradientRMS_M06_Ch06 > 50; AspectRatio_M06> = 0.85. Thresholds set were all based on the brightfield channel (Channel 6).

### Quantification of γH2AX foci

Several preprocessing steps were performed prior to analyzing the cell images. Cell images, captured using the ImageStream, were converted from the .rif format to .cif files using the IDEAS software (v6.2; Amnis), and the image metadata was exported in plaintext (.txt). Using the Bio Formats plugin^39^ for Matlab (Mathworks), .cif files were then analyzed to produce .tif imagestacks of identical size by taking the 99th percentile of image sizes and cropping larger images, and extending smaller images with pixel values that matched the background and noise level of the image. Image data were then analyzed locally or processed via a Jupyter Google Colab notebook as described below.

In the Jupyter Google Colab notebook, images were cropped to 64 × 64 pixels around the center of mass of the cell in each image. Next, the pixel intensity values were normalized [0,1], as it has been proven empirically to improve the performance of the neural network. These normalized images served as the input to a deep neural network trained to filter the cell background and other noise sources in order to detect foci associated with DSB.

Image-analysis applications lacking a ground truth pose a challenge for neural network training, since the data are not labeled (i.e., we do not know the true number of DSBs in every cell image). While a manually tagged set of data can be used for training, creating a large data set of cells is cumbersome and may not be broadly applicable. Here, we use a hybrid approach of augmenting cell-image data without foci to generate a larger labeled training data set for the neural network.

The training set was generated from 200 images of untreated cells, which were verified to contain zero foci. For each training image, we added a random number of spots in the range [0,10] to create a simulated observation image. We repeated this step to generate 1,600 unique training samples for the training phase. Each simulated spot was a 2D Gaussian parametrized according to the formula:

$$\left(1\right)Foci\left(x,y\right)=\frac{A}{2\pi \sigma^{2}}e^{\frac{-{\left(x-x_{0}\right)}^{2}-{\left(y-y_{0}\right)}^{2}}{2\sigma^{2}}}$$

where x,y marks the image pixels, $x_{0},y_{0}$ marks the ground truth location of the focus, σ marks the standard deviation of the Gaussian randomly chosen in the range [0.75, 2.25] pixels, and *A* is the amplitude, which is equal to $3\cdot \sqrt{\sigma^{3}}$. These parameters were chosen empirically as they were proven to provide sufficient signal-to-noise ratio for detection, but not too high a signal-to-noise ratio that would hinder the network training rate. The observations of the neural network are these augmented cell images.

After data augmentation, we trained the neural network on a standard workstation equipped with 16 GB of memory, an Intel^®^ Core™ i7—8700, 3.20 GHz CPU in less than 3 h. We used U-net network architecture^40^ with six convolutional layers. Each layer is followed by a batch normalization layer and parametric rectified linear unit (PReLU) layer with skip connections between layers of the same size. The input to the network is the augmented cell image of size 64 × 64 pixels, and the output is the image containing 2D Gaussians of the same size. We trained the network using mean squared error loss term and ADAM optimizer^41^ with a learning rate of 10^–5^ and betas = (0.99, 0.999) for 250 epochs. The trained model weights are supplied in the github page of this work (https://github.com/alonsaguy/Rapid-CRISPR-Quantification).

For testing, we supply an analysis pipeline written in Python in a Jupyter Google Colab notebook. This pipeline quantifies the number of foci and their intensities based on .tif images generated after the first preprocessing steps. The main steps of the notebook are: (1) mounting the notebook on a Google Drive account and installing Python dependencies; (2) loading the pretrained neural network weights; (3) passing the input through the neural network and analyzing the output images; (4) plotting each focus and cell intensity estimate and finally exporting the data to different software (e.g., Microsoft Excel or Matlab).

The analysis of step 3 is based on previous work for single molecule localization with high resolution in dense emitter areas.^42^ First, we draw a mask of possible foci locations by observing pixels with intensity higher than the 85th percentile of the image pixel intensity. Next, we take a patch of 9 × 9 pixels around each possible focus and fit a 2D Gaussian according to equation (1). If the *R*^2^ fitting score is less than a threshold of 0.85, we assume the analyzed pixel is discarded. Otherwise, we calculate a confidence level for each focus based on the following formula:


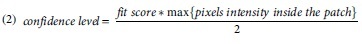


Localizations are discarded for two reasons. First, if the confidence level of a possible focus is lower than a threshold of 100, it is discarded. A possible focus with a confidence level higher than this threshold is considered as a new focus, and its location is saved as the first output of the algorithm. Second, if the intensity falls below a threshold of 100 integrated counts, it is discarded. This helps remove spurious fluctuations in the cell background from being counted. Any focus falling below the intensity threshold was still counted in the focus intensity metric, where they are intrinsically deweighted.

### GUIDE-Seq

The GUIDE-seq method was used to identify global editing events in an unbiased fashion for the *VEGFA* gRNA in order to create a comprehensive primer panel for rhAmpSeq multiplex polymerase chain reaction (PCR; GUIDE-seq for *RAG1* and *RAG2* was conducted by our group and reported previously^30^ and the FASTQ files were downloaded for additional analysis from the sequence read archive [SRA] under accession number PRJNA628100; Supplementary Data S1).

The procedure was performed with HEK293-Cas9 cells, which were electroporated with *VEGFA* two-part gRNA system (10 μM) along with dsODNs, a 34 bp dsDNA donor fragment (see Tsai *et al.*^26^). Then, the cells were reconstituted in SF cell line electroporation solution according to the manufacturer's protocol (Lonza). The cells were placed in the Lonza 4D-Nucleofector and electroporated using the CM-130 program. Seventy-two hours after electroporation, genomic DNA (gDNA) was extracted from the cells by column purification. NGS library preparation, sequencing, and operation of the GUIDE-seq software were performed, as described previously.^26,30^

The genomic locations of the GUIDE-seq-identified off-target sites (OTEs) were analyzed via the University of California, Santa Cruz (UCSC), genome browser version GRCh38/hg38. The FASTQ files for the GUIDE-seq data for *VEGFA* were uploaded to the SRA under accession number PRJNA762559.

### rhAmpSeq

rhAmpSeq primer amplification depends on perfect-match hybridization to the target DNA and subsequent recognition of the RNA/DNA hybrid duplex by RNase H2 to cleave the target-matched primers that contain an RNA base and polymerase-blocking modification and allow for locus-specific amplification.^43^ Primers were designed to flank the on-target and all off-target sites for the *VEGFA* gRNA, as nominated by GUIDE-seq in HEK293-Cas9 cells, following editing with the two-part gRNA system. Primers were pooled for locus-specific rhAmpSeq, followed by a universal PCR to add adaptor ends for NGS. PCR amplicons were sequenced on an Illumina MiSeq instrument (v2 chemistry, 150 bp paired end reads). The read depth was at least 3,000 reads per locus. The average read depth was around 40,000 reads for each gRNA.

Data were analysed using the CRISPECTOR software tool, as described previously,^31^ to produce inferred activity rates at the analyzed sites. The rhAmpSeq data for *RAG1* and *RAG2* were previously published, and the FASTQ files were downloaded for additional analysis from the SRA under accession number PRJNA630002. The FASTQ files for the rhAmpSeq data for *VEGFA* and a second repeat for *RAG1* were uploaded to the SRA under accession number PRJNA762559.

## Results

### High-throughput visualization of CRISPR-Cas9-induced DNA damage in CD34^+^ HSPCs

Employing IFC to acquire thousands of cell images, we characterized the time course of CRISPR-induced DDR in CD34^+^ HSPCs (for a detailed protocol, see Methods).

**FIG. 1.**
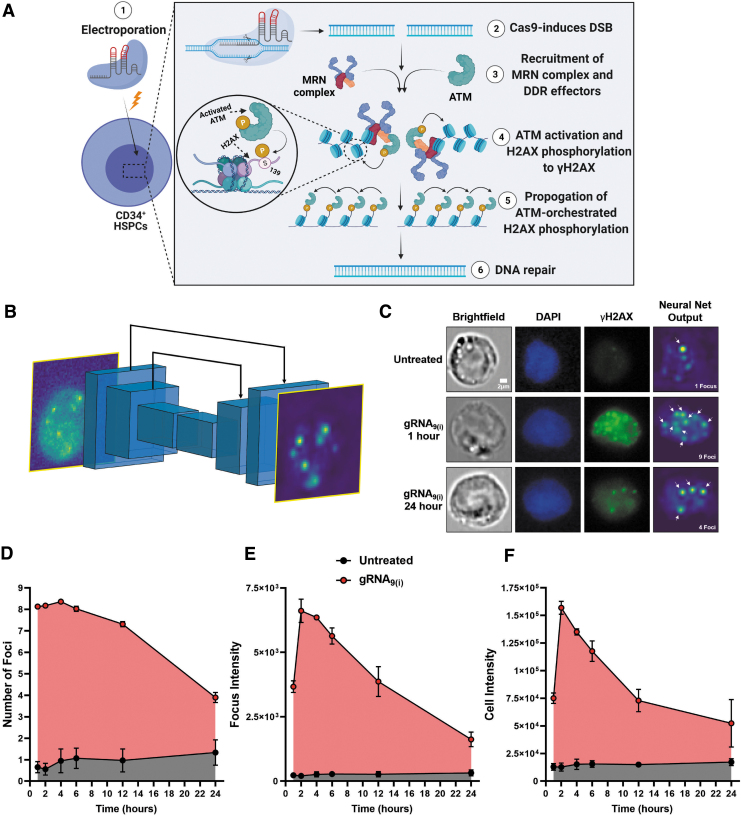
High-throughput characterization of CRISPR-induced DNA damage response. **(A)** Schematic of CRISPR-mediated gene editing of human CD34^+^ HSPCs cells. (1) Cells are electroporated with the ribonucleoprotein complex, which is (2) shuttled into the nucleus where it interacts with the genome. (3) When a DSB occurs in the DNA of mammalian cells (e.g., due to CRISPR-Cas9), a three-protein complex called the MRN complex senses the open DNA ends and then recruits and activates ATM protein to the break site. This leads to (4) phosphorylation of H2AX in the flanking nucleosomes, triggering the DNA damage response pathway. The phosphorylated variant of H2AX, γH2AX, binds to other repair factors, creating a feed-forward loop that (5) propagates activated ATM and γH2AX over vast chromatin domains signaling for (6) DNA repair. **(B)** Neural network architecture composed of six convolutional layers (see Methods). The input to the net is a cell image, and the output is a heatmap that filters the cell background and highlights foci. **(C)** Representative brightfield, DAPI (blue), γH2AX (green) images, and the neural net output. Representative images of untreated cells and cells treated with gRNA_9(i)_ at 1 h and 24 h post electroporation. Scale bar: 2 μm. **(D)**–**(F)** 24 h time course of gRNA_9(i)_ (red) and untreated (black) cells (*n* = 3). Data points represent the mean, and error bars represent SEM. Focus and cell intensity are listed in AU. **(D)** Average number of foci. **(E)** Average focus intensity (the integrated signal of all foci per cell). **(F)** Average cell intensity. HSPCs, hematopoietic stem and progenitor cells; DSB, double-strand break; ATM, ataxia telangiectasia mutated; H2AX, histone 2AX; SEM, standard error of the mean; AU, arbitrary units.

**FIG. 2.**
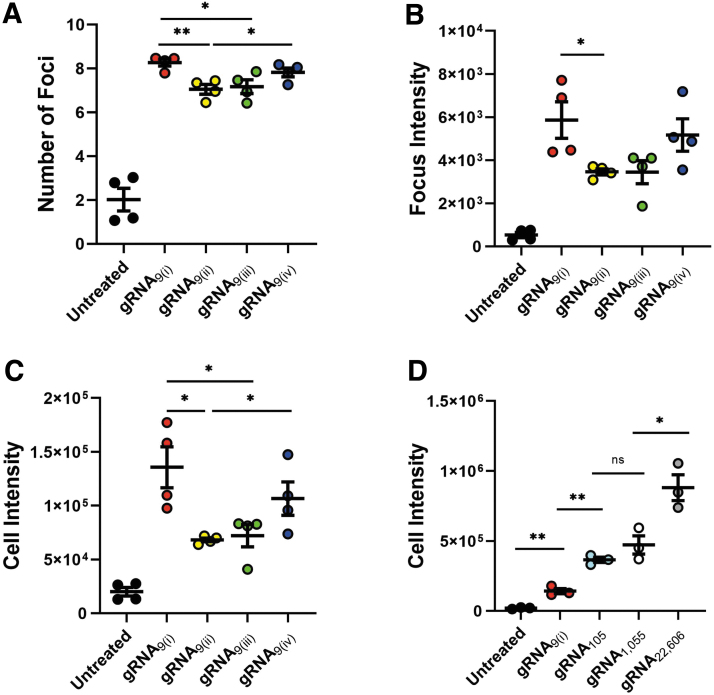
CRISPR-induced DNA damage by gRNAs with multiple on-target sites. **(A)**–**(C)** Average number of foci, focus intensity, and cell intensity 4 h after electroporation for cells edited with gRNAs each containing nine on-target sites in the genome (*n* = 4). **(D)** Average cell intensity for gRNA_9(i)_ (nine on-target sites in the genome)_,_ gRNA_105_ (105 on-targets)_,_ gRNA_1,055_ (1,055 on-targets)_,_ and gRNA_22,606_ (22,606 on-targets) 4 h after electroporation (*n* = 3). Error bars represent SEM. *p < 0.05; **p < 0.005 (*t*-test). Focus and cell intensities are listed in AU. gRNA, guide RNA.

In brief, (1) the CRISPR-Cas9 RNP complex was delivered into human CD34^+^ HSPCs by electroporation (Fig. 1A); (2) cells were fixed and immunostained with fluorescently labeled anti-γH2AX antibodies to visualize DSB sites^44^; (3) samples were loaded and imaged in the Amnis ImageStream IFC; (4) typically 1,000 or more objects were imaged per sample (mean of 1,560) over the course of 5–10 min, and cell images were exported and (5) processed with a neural network to suppress background and identify candidate foci, which were localized, thresholded, and counted (Fig. 1B). After filtering for defocused and/or partially cropped cell images, an average of 500 cell images remained to be analyzed per sample.

Cell populations were followed for 24 h after electroporation (*t* = 0) and were compared to cells electroporated without any RNP complex (herein referred to as untreated; Fig. 1C–F).

To measure the dynamics of the DDR in CRISPR-treated cells, we used a profligate gRNA, with nine on-target sites throughout the genome, gRNA_9(i)_ (see Table 1 for sequence). γH2AX foci were observed at the earliest measured time point post electroporation (1 h) and persisted throughout the 24 h observation window, peaking at 4 h (mean number of foci per cell of 8.7; Fig. 1D and Supplementary Fig. S1A). Cells in the untreated population showed significantly less DNA damage at all time points. However, some foci were nonetheless observed in 56 ± 15% of cells, with an average of ∼1 focus per cell (Fig. 1D), possibly due to spontaneous DSBs that have been reported previously.^45^

In addition to counting the number of foci per cell, we parameterized the integrated fluorescent signal from all foci per cell, herein referred to as the focus intensity (Fig. 1E). This parameter can mitigate the effect of false-positive counts due to spurious dim spots by weighing the value of each focus by its brightness. Finally, we characterized the total γH2AX fluorescence intensities of each cell, which is equivalent to a standard flow cytometry measurement, and is referred to as cell intensity (Fig. 1F). While the measurement of average cell intensity lacks subcellular detail, it does not require parameter tuning to separate fluorescence background from signal.

### Higher CRISPR-Cas9 promiscuity induces greater DDR

To characterize the difference between gRNAs with the same number of on-target sites, we screened four gRNAs with nine on-target sites each: gRNA_9(i)_, gRNA_9(ii)_, gRNA_9(iii)_, and gRNA_9(iv)_ (Table 1). Four hours after electroporation, these gRNAs had highly repeatable measurements for the number of foci (7–9; Fig. 2A). However, the focus intensity and cell intensity were relatively more heterogeneous (Fig. 2B and C and Supplementary Fig. S2A). This indicated that not all CRISPR-induced DSBs and the subsequent DDR signals are equivalent, thus motivating cell imaging. Additionally, the average relative error within each treatment is lower in the number of foci compared to the focus- and cell-intensity measurements, respectively (6%, 24%, and 23%).

To test how γH2AX staining functions over a much broader scale of DNA damage, four gRNAs—gRNA_9(i)_, gRNA_105_, gRNA_1,055_, and gRNA_22,606_—with nine on-targets sites, 105 on-targets sites, 1,055 on-targets sites, and 22,606 on-targets sites were tested (Table 1).

At high levels of DNA damage, images cease to contain resolvable foci, and thus only cell intensity remains a relevant measure (Fig. 2D and Supplementary Fig. S2B–D). To determine if 3D confocal microsocopy could further resolve foci in cell images, we recorded several cells by 3D confocal microscopy (Supplementary Movie S1). While individual foci were visible for gRNA_9(i)_, individual repair centers in higher-damage conditions were still not resolvable. We therefore conclude that subcellular imaging is best suited for detecting lower levels of DNA damage.

### Different gRNAs have distinct DDR dynamics

While CRISPR-Cas9-induced DDR with gRNAs that have multiple on-target sites has been shown previously,^16^ a much more challenging task that is relevant for deploying CRISPR in the clinic is comparing the DDR effects of gRNAs with only a single on-target site characterized by low levels of DSBs. Since CRISPR-Cas9 is known to elicit a DDR,^46^ which is thought to be at least partially due to off-target activity, it is critical to identify a gRNA with the most specific editing possible. We therefore set out to test several gRNAs with only one on-target site in the genome, selecting three gRNAs with well-characterized on- and off-target activity (herein referred to as *RAG1*, *RAG2*, and *VEGFA*; Table 1).

**FIG. 3.**
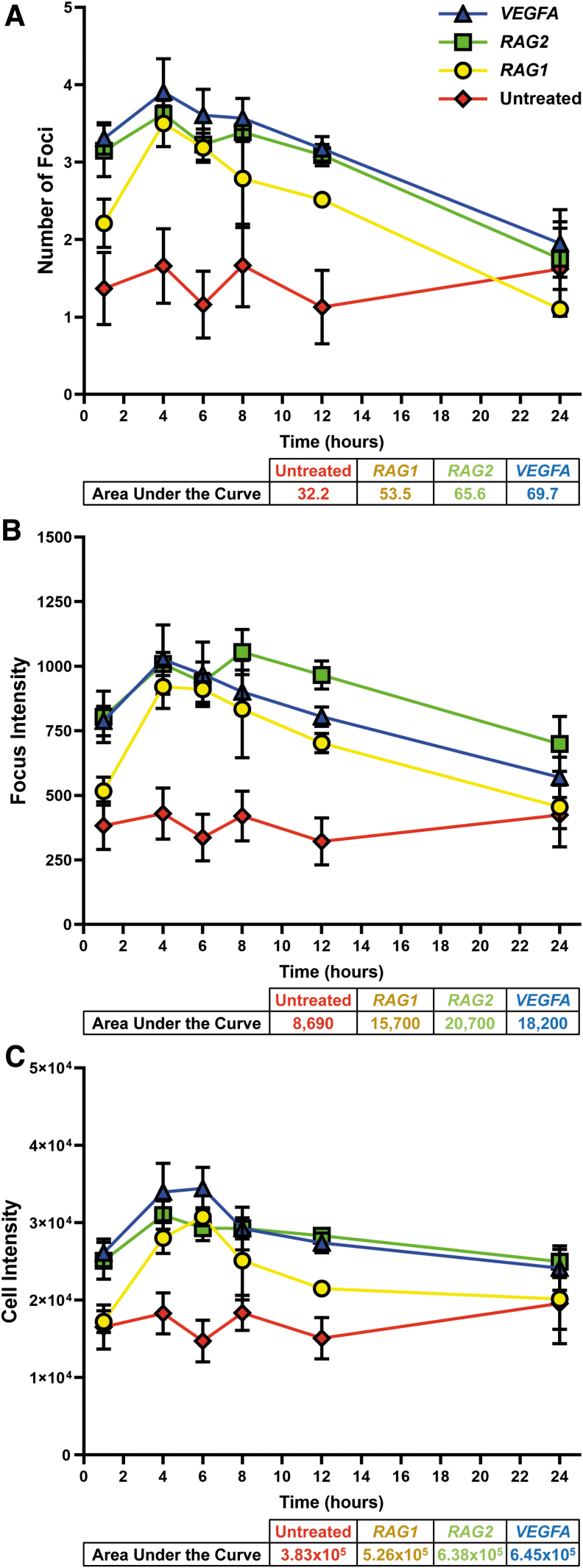
Quantification of single-on-target gRNAs DNA damage and repair dynamics. Time course for gRNAs with a single on-target. For time points 1, 6, and 12 h, *n* = 6; for time points 4, 8, and 24 h, *n* = 4. Data points represent the mean, and error bars represent SEM. **(A)**–**(C)** Average number of foci, focus intensity, and cell intensity. Focus and cell intensities are listed in AU.

We electroporated CD34^+^ HSPCs with these gRNAs individually to induce site-specific editing and measured γH2AX staining to detect the levels of DDR over a 24 h window. Similar to the multi-on-target gRNAs, the formation of γH2AX foci was observed at the earliest measured time point, 1 h post electroporation (Fig. 3A). The significant number of foci relative to the control group persisted through 12 h, returning to baseline by the 24 h time point. The peak in the γH2AX focus count was observed between 4 and 8 h (Fig. 3A; individual measurements shown in Supplementary Fig. S3).

Interestingly, *RAG2* and *VEGFA* gRNAs both showed faster and more persistent DDR signaling relative to *RAG1*. After 1 h, *RAG2* and *VEGFA* showed a higher number of foci and higher γH2AX staining intensity than *RAG1* (Fig. 3A). After 12 h, we observed that the number of foci, focus intensity, and cell intensity had all decreased more for *RAG1* than for *RAG2* and *VEGFA* relative to their peaks (Fig. 3B and C). In terms of the integrated area under the curve for the 24 h window, the total detected DDR for *RAG1* was substantially lower than for *RAG2* and *VEGFA*, yet still significantly above the background (Fig. 3A–C), indicating a difference in cutting efficiency.

### Image-based profiles with greater DDR were observed for gRNAs with higher total CRISPR-Cas9 editing

**FIG. 4.**
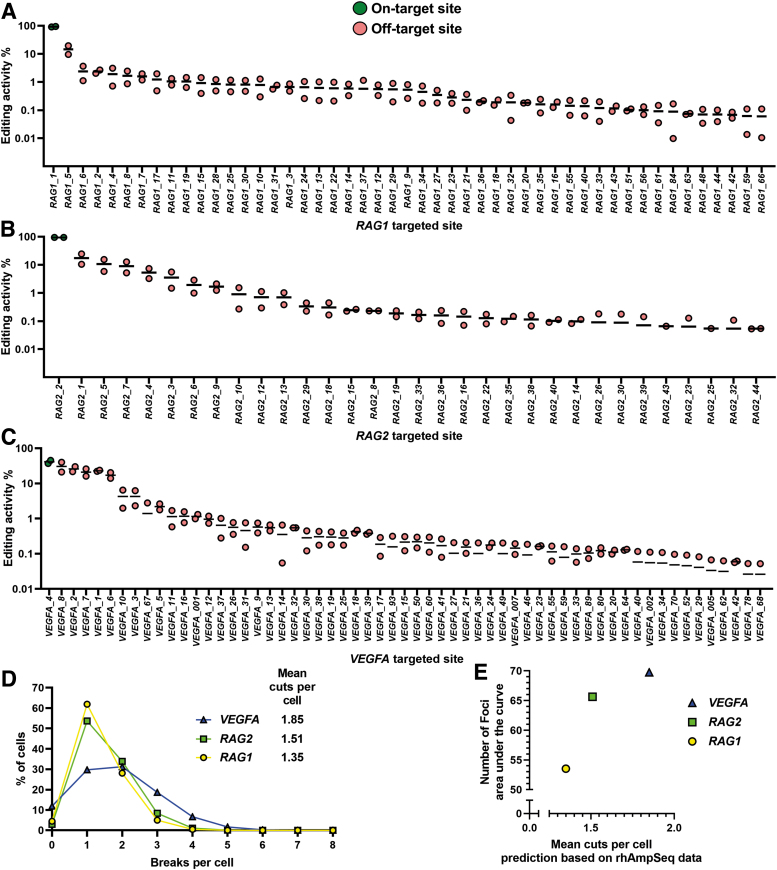
Characterization of single-on-target gRNAs with next-generation sequencing. **(A)**–**(C)** On- and off-target rhAmpSeq data analyzed with CRISPECTOR, cutoff at 0.05% editing efficiency (*n* = 2). Details for primer panel for rhAmpSeq nominated by GUIDE-seq found in Supplementary Data S1. Bars represent the mean efficiencies. If the site was only detected in one repeat, the undetected value is determined to be 0. Performed in HEK293-Cas9 cells. **(A)** *RAG1* (primer panel of 84 detected sites nominated by GUIDE-seq and a sum of 134.7% total editing activity) from Shapiro *et al*.^30^ **(B)** *RAG2* (50 sites, sum of 149.6%) from Shapiro *et al*.^30^ **(C)** *VEGFA* (105 sites, sum of 183.9%). **(D)** Simulated distribution of the number of expected break sites per cell based on the editing efficiencies determined by rhAmpSeq analysis. **(E)** Integrated area under the curve for the number of foci over the time course (Fig. 3A) versus mean cuts per cell estimated from the rhAmpSeq-determined editing efficiencies for *RAG1*, *RAG2,* and *VEGFA* gRNAs.

To elucidate how the differences in the measured DDR patterns relate to gene-editing outcomes for these three gRNAs, we characterized the on- and off-target editing efficiencies using NGS. Our group and others have utilized GUIDE-seq to identify and report on the off-target activity for gRNAs that target the *RAG1* and *RAG2* genes^30^ and the *VEGFA* gene.^26,29,47^ Based on GUIDE-seq analysis, we designed a rhAmpSeq multiplex PCR panel to measure on- and off-target editing efficiencies. The NGS rhAmpSeq data were analysed with the CRISPECTOR tool^31,48^ to contextualize our findings from the Imagestream (Fig. 4A–C).

Interestingly, the summed probability of gene-editing efficiency was higher in the samples where the imaging-based results in terms of the integrated curve describing the average number of foci detected were greater (*RAG1* producing a lower DDR than that of *RAG2* and *VEGFA*). The DDR patterns of *RAG2* and *VEGFA*, however, were markedly similar to one another. It is interesting to note that despite *RAG2* having an on-target efficiency of 94.8% (Fig. 4B) and *VEGFA* on-target efficiency being only 41.3% (Fig. 4C), the high levels of off-target editing by *VEGFA* supplement the overall damage signal to produce a larger DDR than *RAG2*, as shown by a simulated number of cut sites based on the measured editing efficiency (Fig. 4D).

By showing that gRNAs with higher genome-editing activity, as determined by the rhAmpSeq data, also produce a greater DDR, as observed in our image-based profiles (Fig. 4E), we indicate that our imaging pipeline is sufficiently sensitive to detect subtle differences in DNA damage patterns between single on-target gRNAs. Therefore, we believe our method can provide a useful, low-cost screening tool to compare a large number of proposed gRNAs rapidly to eliminate those with high levels of genotoxicity that stems in part from significant off-target interactions.

### IFC characterization of rAAV6 treatments for minimizing downstream toxic effects

**FIG. 5.**
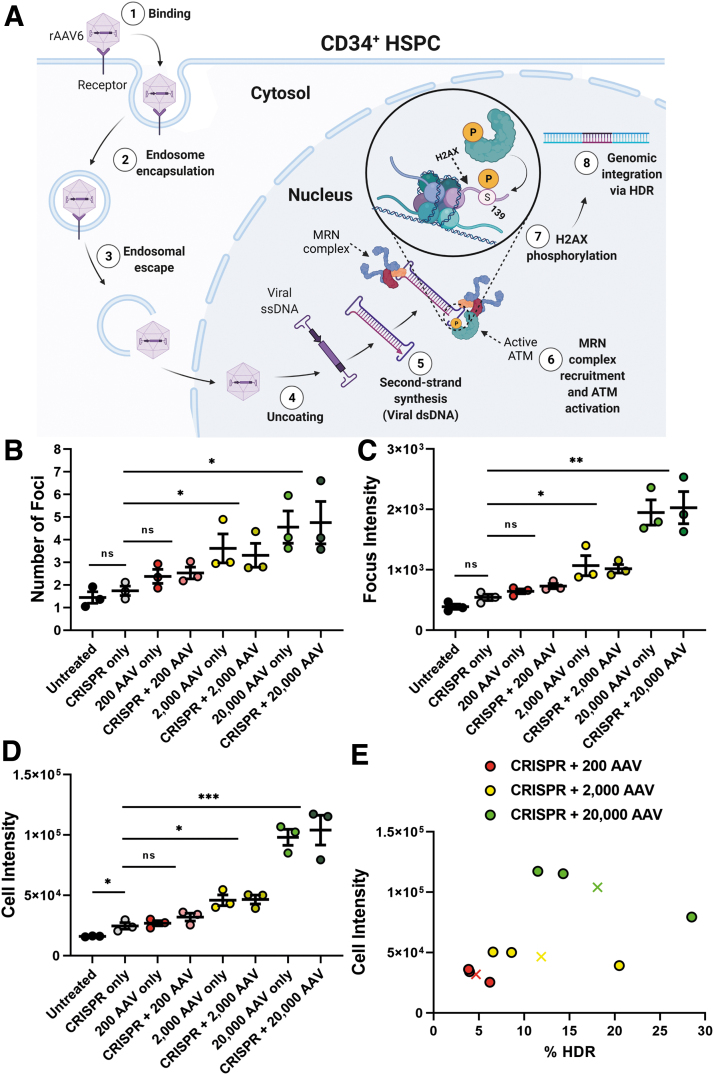
AAV6 treatment causes DDR quantifiable through γH2AX staining. **(A)** Schematic of rAAV6 transduction. The rAAV6 capsid (1) binds and (2) enters the cell. It is transported via an endosomal pathway to the perinuclear region where it is (3) released from the endosome and shuttled into the nucleus through the nuclear pore complex. (4) Once in the nucleus, the virus sheds its protein coat and releases its ssDNA payload. (5) As the ssDNA undergoes second-strand synthesis to dsDNA, the MRN complex is recruited to the viral genome's ITRs and limits the activity of the virus. (6) The MRN in turn activates ATM, which (7) phosphorylates H2AX. (8) The dsDNA can then undergo integration into the genome through the HDR or homologous recombination repair pathway. **(B)**–**(D)** Average number of foci, focus intensity, and cell intensity 48 h after electroporation (*n* = 3). Error bars represent SEM. Focus and cell intensities are listed in AU. **(E)** Average HDR efficiency for each MOIs (200 VG/cell: 4.7%; 2,000 VG/cell: 11.9%; 20,000 VG/cell: 18.1%), as measured by standard flow cytometry (*n* = 3). Cells were stained for tNGFR expression (the reporter gene expressed in the donor DNA). These values are plotted against the average cell intensity for each treatment. Xs indicate mean values for HDR and average cell intensity for each of the three treatment groups. AAV6, adeno-associated virus serotype 6; DDR, DNA damage response; ITR, inverted terminal repeat; HDR, homology-directed repair; MOI: multiplicity of infection; VG, viral genomes.

In addition to detecting CRISPR-induced DDR, our system can report on the genotoxicity induced by other genome-editing components. To demonstrate this, we applied our method to determine the optimal rAAV6 concentration for donor DNA delivery in order to reduce the potentially deleterious DDR while still maintaining clinically relevant levels of HDR. rAAV6 toxicity is a major concern in gene therapy applications, since cells treated with the viruses show heightened DDR signaling^32^ (Fig. 5A). This effect has been associated with reduced cell proliferation and apoptosis, which can pose major issues when transplanting a patient's cells as an autologous stem-cell transplant.^32,34^

We tested a donor for HDR at three MOIs (20,000, 2,000, and 200 VG/cell). The donor DNA design was based on previous CRISPR-Cas9/rAAV6 gene-editing strategies.^49,50^ For each individual MOI, no difference in γH2AX signaling was observed between cells that were treated with only the virus or together with the RNP complex (Fig. 5B–D). This indicated that the DDR signaling comes primarily from episomal rAAV6.^33,51,52^ When using MOIs of 20,000 and 2,000 VG/cell, the DDR was found to be significantly greater than in the control groups, whereas the MOI of 200 VG/cell was comparable to that of the background (Fig. 5B–D and Supplementary Fig. S4A). At high MOIs, we observed pan-nuclear staining, indicating that the high concentration of episomal rAAV6 intermediates may dominate, and therefore mask, genomic DDR.^53^

When we confirmed the level of HDR through flow cytometry, we found that although the efficiency of the integration for 200 VG/cell was lower (4.6%) than that of the two higher MOIs (2,000 VG/cell: 11.9%; 20,000 VG/cell: 18.1%), it still induced effective HDR^54^ (Fig. 5E and Supplementary Fig. S4B). The application of our method for DDR quantification can be an effective way to optimize rAAV6 donor MOIs in an attempt to minimize its downstream toxic effects when used for therapeutic applications.

## Discussion

In this study, we have shown the applicability of IFC to characterize gene-editing-induced DDR. By immunostaining γH2AX and tracking three parameters in cell images—the number of foci, integrated focus intensity, and cell intensity—we quantified the DDR for CRISPR-Cas9 and rAAV6 treatments in HSPCs. Compared to traditional flow cytometry, IFC provides subcellular detail.

The damage response was found to track the number of on-target sites over a broad scale. In samples with relatively high levels of DNA damage, individual foci become obscured by the high density of spots, and the utility of subcellular details is obscured by the finite resolution of the imaging system (Supplementary Fig. S1B). In these situations, cell intensity provides a better description of the cell. At the most clinically optimal regime, where gRNAs are as specific as possible, where DNA damage is kept at a minimum, we found that focus counting provides the best signal-to-noise ratio.

Many techniques exist for counting fluorescently labeled γH2AX foci in images.^13,37^ However, varying degrees of parameter tuning are required to handle the fluorescent background properly. Here, to improve the separation of signal from background, we have harnessed the flexibility of neural networks, which have been shown by our group and others to be effective tools in localization microscopy.^42,55–57^ The input is an individual cell image, and the output is a heatmap that emphasizes the foci in the image while supressing background noise. This heatmap image is used to identify peaks that are then analyzed in original image to extract their intensities (Supplementary Fig. S1C).

To facilitate making this pipeline accessible,^58^ the code is executable in a Jupyter notebook mounted on the Google Colab platform (https://github.com/alonsaguy/Rapid-CRISPR-Quantification). Importantly, this workflow is easily modifiable to add additional steps such as 3D localization^59^ and multicolor imaging^37^ that can be used to colocalize specific CRISPR components.^60^

For both the Imagestream and 3D confocal microscopy, the hundreds of nanometer resolution achieved is insufficient to identify foci in cells under high-damage conditions (Supplementary Movie S1). To significantly expand the upper range for focus counting, super-resolution imaging techniques, such as (f)PALM/STORM,^61–63^ can and have been utilized to characterize DSB repair with sub-focus resolution^11,48^ at a significant cost to imaging time.

Flow-based imaging provides a high-throughput alternative to conventional microscopy. For naturally nonadherent cells, such as the HSPCs utilized in this study, no additional steps to adhere the cell to the surface are required. The high throughput afforded by flow-based imaging is therefore particularly advantageous for characterizing heterogeneous systems such as CRISPR- and rAAV6-treated cells.

In this study, we quantified the genotoxic effects of individual gene-editing components after treatment. Specifically, we measured the DDR as a result of CRISPR-Cas9 editing and rAAV6 treatment. Intriguingly, intensity measurements exhibited high variability even within treatments (Fig. 2B and C). This may be due to the extent of amplification and the locus-specific pattern of γH2AX that can spread over megabases from the cut site.^10^ The overall labeling efficiency may therefore be sensitive to the chromatin structure surrounding the cut site, consistent with a growing body of evidence showing that (1) the genomic topology has a substantial effect on repair protein recruitment,^64–66^ and (2) the distribution of the γH2AX signal can be asymmetric around the DSB, with lower density around transcribed regions.^67,68^

When the DNA damage is extremely high (e.g., with many target sites), the cell intensity measurements were found to be a more robust parameter, since individual foci were unresolvable (Fig. 2D and Supplementary Fig. S2B–D), although these gRNAs are less clinically interesting. Therefore, to attain the largest dynamic range, it is best to utilize all three metrics.

To maximize CRISPR's site-specific capabilities, clinicians and researchers are looking to develop methodologies to search for gRNAs with only one on-target and as few off-targets as possible to avoid deleterious downstream effects. As a proof of concept for this pipeline, we chose three gRNAs with on- and off-target capabilities that have been tested previously.^26,29,30,47^

As much as 12 h post electroporation, these gRNAs showed a significant DDR above background levels (Fig. 3 and Supplementary Fig. S3E and G–I). Compared to *RAG1*, the dynamics of DDR measured in *VEGFA* and *RAG2* showed a faster rise and slower fall—in terms of the percentage of cells with a large number of foci, five or more. At the 8 h time point, a larger difference was detected (*RAG1*: 25.2%; *RAG2*: 31.2%; *VEGFA*: 40.4%) compared to the control (untreated: 10.6%; Supplementary Fig. S3D). This indicates that although the majority of Cas9-induced cutting occurs within the first 2 h,^69^ a significant DDR persists well beyond that time, which could be explained by the presence of unrepaired DSBs, which is undesirable.^20^

When compared to the NGS data of all edited sites, the sequencing profiles of *RAG1* and *RAG2* are similar, namely high on-target editing efficiency (93.0% and 94.8%, respectively), and a large number of off-target sites with low editing efficiency. In terms of expected editing, this makes it is very likely for a given cell to have at least one edited site. In contrast, *VEGFA* produces markedly lower (41.3%) on-target editing efficiency than *RAG1* and *RAG2* yet has several off-target sites with relatively high editing efficiency, producing a broader distribution of expected cut sites. Here, by quantifying the number of foci in a large number of cells, we can resolve the subtle differences in the average (Fig. 4A–D).

The effect of the high levels of off-target editing by *VEGFA* supplement the lower on-target editing efficiency to produce an overall DDR larger than that of *RAG1* and *RAG2* both in our simulation and in our image-based analysis (Fig. 4E).

When CRISPR-Cas9 is utilized as a tool for gene correction or gene insertion, a donor DNA, often delivered by viral vectors, must be used. For *ex vivo* genome-editing applications, where edited cells will be transplanted back into the patient, reduction of virus-induced toxicity is critical. The challenge is therefore optimizing a protocol that minimizes viral load to promote cell viability and simultaneously delivers enough gene-edited cells to ensure succesful engraftment and therapy. In monogenic disorders such as severe combined immunodeficiency in which patients are born unable to develop functional immune systems, correcting HSPCs can generate healthy cells, capable of differentiation, with a selective advantage over the defective host cells. As a result, a small number of corrected cells is enough to achieve therapeutic outcomes.^70^

Our results show that a higher MOI produces a more robust γH2AX signal, and it is therefore possible to utilize the DDR to optimize MOI to maximize cell viability (low DDR) while achieving a sufficiently high HDR to reconstitute the immune system (Fig. 5B–E).

## Conclusions

New gene therapies using CRISPR-Cas9 and AAV technologies must be validated to ensure that *ex vivo* editing is unlikely to lead to the accumulation of mutations and downstream complications. This is also true for potential therapies using multiplexed CRISPR experiments, namely the use of multiple gRNAs simultaneously. For this reason, a tool that can inexpensively and accurately screen potential gRNAs or gRNA combinations and test multiple MOIs is extremely valuable. While future work will help elucidate the genome-editing-induced DDR mechanisms, our data show that IFC can quickly evaluate the genotoxicity of prospective editing treatments—an important step toward optimal deployment in the clinic.

## Supplementary Material

Supplemental data

Supplemental data

Supplemental data

Supplemental data

Supplemental data

Supplemental data
