# Supplementary material for: High-Throughput Imaging of CRISPR- and Recombinant Adeno-Associated Virus–Induced DNA Damage Response in Human Hematopoietic Stem and Progenitor Cells
Source: CRISPR J. 2022 Feb 22;5(1):80–94. doi: 10.1089/crispr.2021.0128 (PMC8892977; doi:10.1089/crispr.2021.0128)
Supplement: Supplemental data [file Suppl_FigureS1.docx]

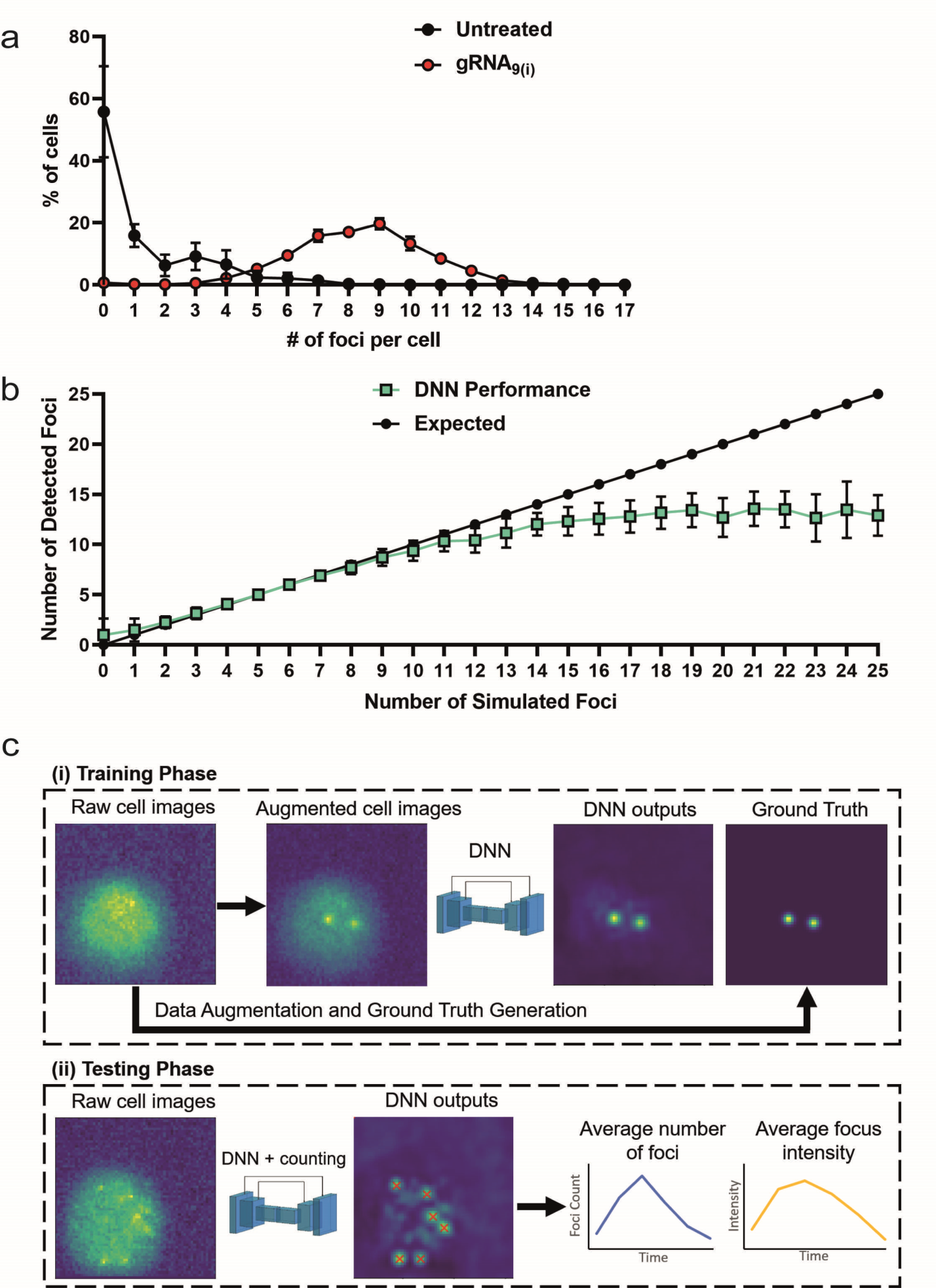


***Supplementary Figure 1: High-throughput characterization of CRISPR-induced DNA damage and analysis via deep neural network.*** (a) The *Number of Foci* measured for experimental images recorded 1 hour post-electroporation for Untreated and gRNA_9(i)._ (b) The *Number of Foci* measured per image based on the deep neural network (DDN) performance (blue) and for simulated foci [0, 25] (black). The error bars represent the standard deviation. (c) Schematic of the DNN. (i) Training phase: augmented cell images are generated by adding a random number of spots [0, 10] to an experimentally acquired cell image. These augmented images are processed by the DNN and compared to an image of the foci without the cell background, i.e. a ground truth. The mean squared error between the outputs of the DNN and the ground truth is used as our loss term for net optimization. (ii) Testing phase: images captured by our microscope to the neural network. The output of the neural network is being processed by our counting algorithm to provide estimates of the *Number of Foci* and intensity per focus. The latter is summed to calculate the *Focus Intensity*.
