# Supplementary material for: High-Throughput Imaging of CRISPR- and Recombinant Adeno-Associated Virus–Induced DNA Damage Response in Human Hematopoietic Stem and Progenitor Cells
Source: CRISPR J. 2022 Feb 22;5(1):80–94. doi: 10.1089/crispr.2021.0128 (PMC8892977; doi:10.1089/crispr.2021.0128)
Supplement: Supplemental data [file Suppl_FigureS2.docx]

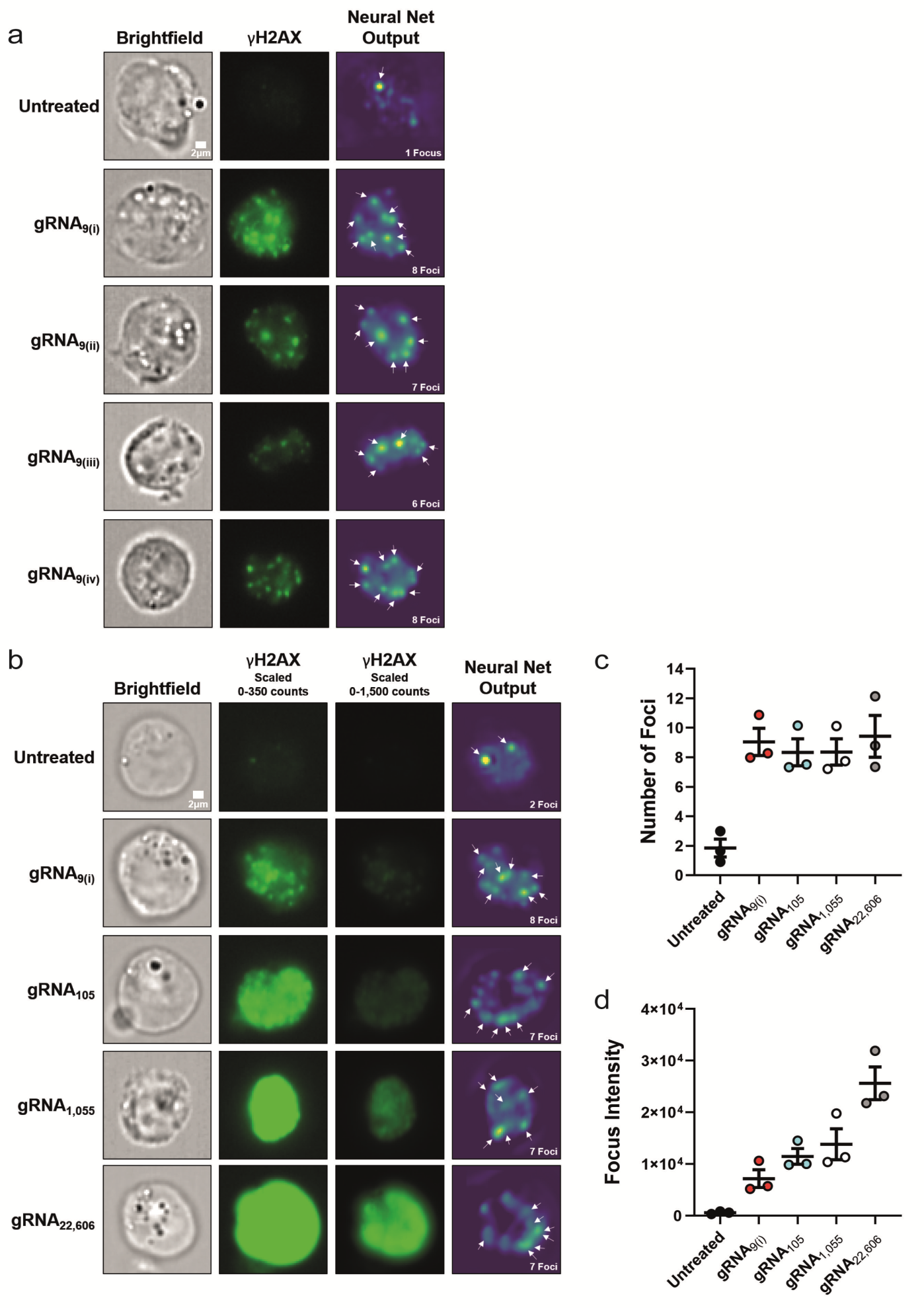


***Supplementary Figure 2: More CRISPR-Cas9 editing produces greater DDR.*** (a) Representative cell images of gRNAs with 9 on-target sites in the genome (gRNA_9(i),_ gRNA_9(ii),_ gRNA_9(iii),_ and gRNA_9(iv)_). (b) Representative cell images of gRNAs with many on-target sites in the genome (gRNA_9(i)_ [9 on-target sites in the genome]_,_ gRNA_105_ [105 on-targets]_,_ gRNA_1,055_ [1,055 on-targets]_,_ and gRNA_22,606_ [22,606 on-targets]). (c-d) *Number of Foci* and *Focus Intensity* corresponding to *(b)* 4 hours after electroporation, (N=3). Error bars represent SEM. *Focus Intensity* is listed in AU. Scale bars: 2μm.
