# Supplementary material for: High-Throughput Imaging of CRISPR- and Recombinant Adeno-Associated Virus–Induced DNA Damage Response in Human Hematopoietic Stem and Progenitor Cells
Source: CRISPR J. 2022 Feb 22;5(1):80–94. doi: 10.1089/crispr.2021.0128 (PMC8892977; doi:10.1089/crispr.2021.0128)
Supplement: Supplemental data [file Suppl_FigureS3.docx]

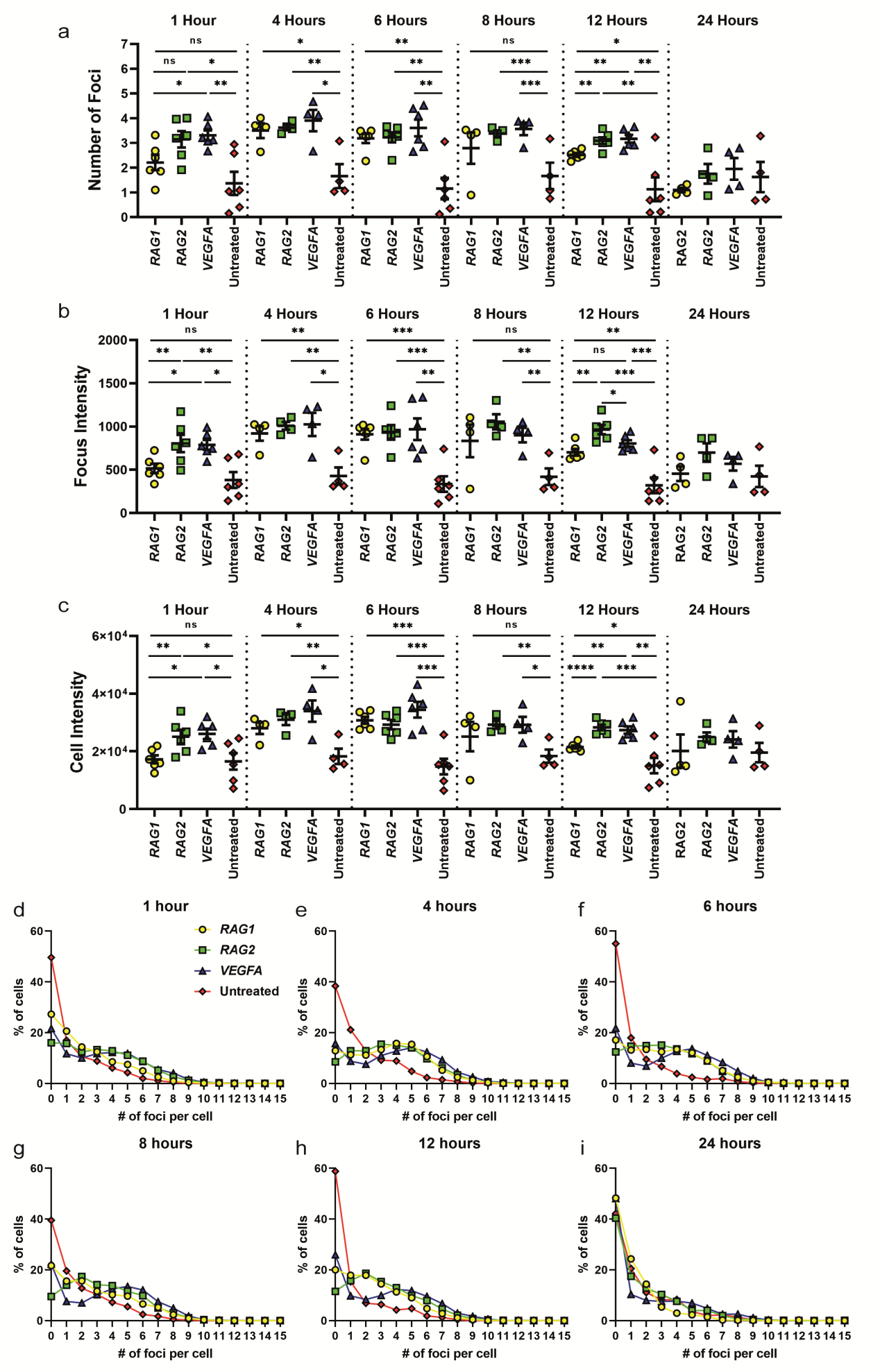


**Supplementary Figure 3: DDR dynamics for one-cut gRNAs.** (a-f) Frequency of Number of Foci over the time course for gRNAs with a single on-target (Samples: RAG1, RAG2, VEGFA, and Untreated cells) For time points 1, 6, and 12 hours, (N=6); for time points 4, 8, and 24 hours, (N=4). (a) 1 hour time-point (b) 4 hours (c) 6 hours (d) 8 hours (e) 12 hours (f) 24 hours. (g-i) Time course for gRNAs with a single on-target for all measured time points (1, 4, 6, 8, 12, and 24 hours). (g-i) Average Number of Foci, Focus Intensity, and Cell Intensity by time point. Focus and Cell Intensities are listed in AU. Error bars represent SEM. * p<0.05, ** p<0.005, *** p<0.0005, and **** p<0.00005 as analyzed by t-test.
