# Supplementary material for: High-Throughput Imaging of CRISPR- and Recombinant Adeno-Associated Virus–Induced DNA Damage Response in Human Hematopoietic Stem and Progenitor Cells
Source: CRISPR J. 2022 Feb 22;5(1):80–94. doi: 10.1089/crispr.2021.0128 (PMC8892977; doi:10.1089/crispr.2021.0128)
Supplement: Supplemental data [file Suppl_FigureS4.docx]

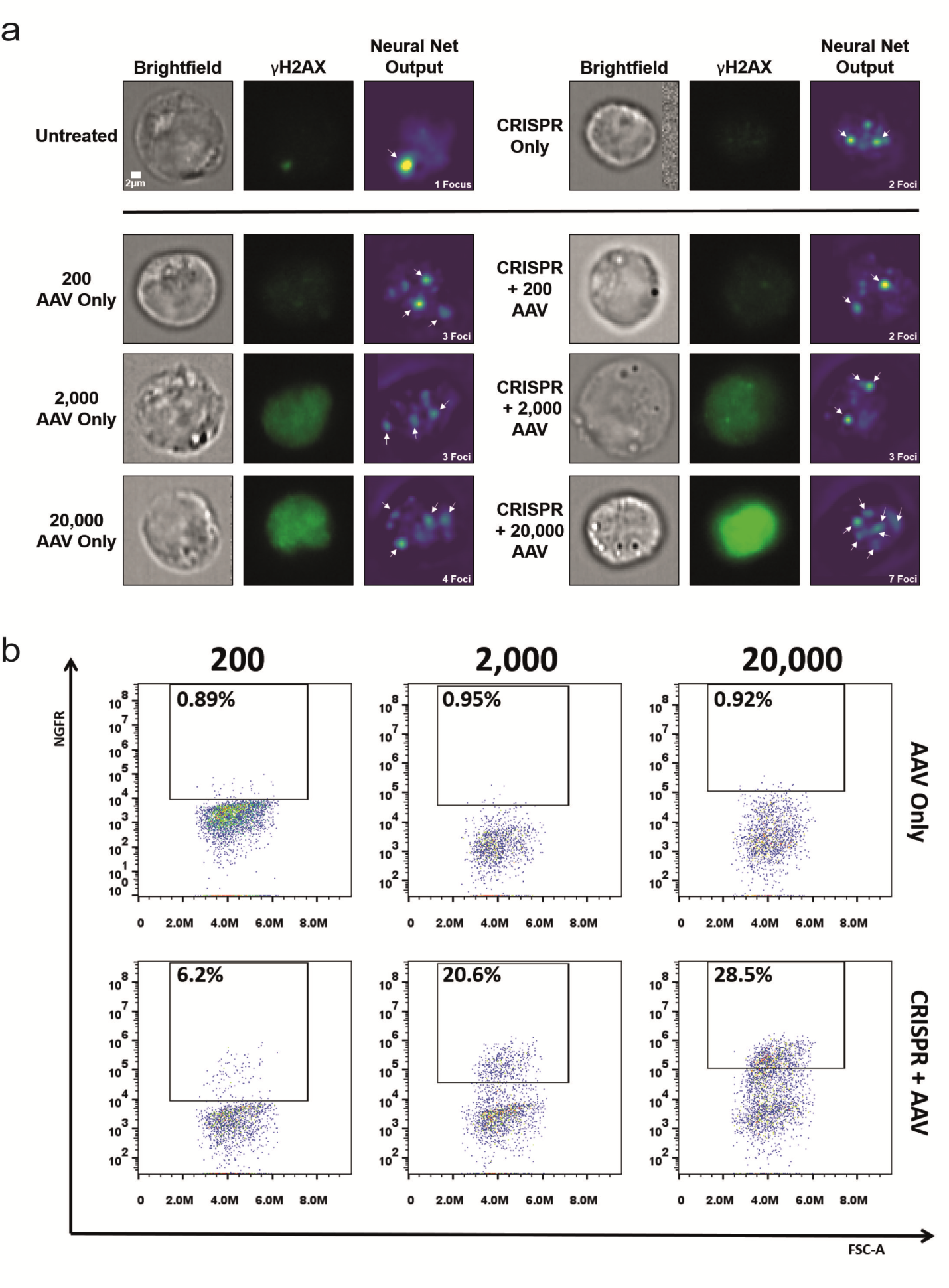


***Supplementary Figure 4: rAAV6-induced DDR quantifiable via γH2AX staining.*** (a) Representative cell images 48 hours post-electroporation. Fluorescence-channel images are displayed with [20, 180] counts, except for 20,000 AAV Only and CRISPR + 20,000 AAV (bottom row) which are displayed with [20, 300] counts. Scale bar: 2μm. (b) *Top row:* Flow cytometry data showing a negative control for each MOI (20,000, 2,000, and 200 VG/cell) to determine population gating. A sample of cells was treated only with the rAAV6 vectors without the RNP, this is to account for the episomal expression of the reporter gene which was determined to be <1%. *Bottom row:* HDR efficiency for each MOI*.* Cells were stained for tNGFR expression (the reporter gene expressed in the donor DNA).
